# Gene Expression of Promyelocytic Leukemia Proteins and IFN-γ Is Reduced in Rotavirus-Infected Children

**DOI:** 10.29252/ibj.24.2.128

**Published:** 2019-10-30

**Authors:** Ahmed Jasim Mohammed, Zeenah Weheed Atwan, Aida Abdul-Kareem

**Affiliations:** Department of Biology, College of Science, University of Basra, Basra, Iraq

**Keywords:** Gene expression, IFN-γ, PML, PML-II, Rotavirus

## Abstract

**Background:**

Rotavirus infection is one of the most common gastroenteritis in the world, and a million cases are registered to enter hospital every year. PMLs are IFN-up-regulated proteins, and one of their critical functions is working as antiviral proteins. Recently, PML-II has been depicted as an isoform responsible for the antiviral function.

**Methods:**

Rotavirus prevalence determination was achieved by PCR and Rapid Adeno/Rota Virus test, while the relative expression assay was carried out by real-time PCR technique. Blood and stool samples were collected from 34 children under five years admitted to the hospital with acute gastroenteritis showing signs of dehydration. RNA samples were extracted from blood specimens and converted to cDNA to be used in gene expression analysis of PML, PML-II, and IFN-γ in rotavirus positive or negative samples.

**Results:**

Rapid Adeno/Rota Virus Antigen Combo Test and PCR assay could detect the virus in stool samples in 45% and 17.6% of cases, respectively. PML in positive samples decreased to 10^4^fold less than the level in negative ones. The same trend was noticed in the level of IFN-γ and PML-II expression as their expression reduced to 10^4^ or 13fold in rotavirus-infected samples compared to the control, respectively.

**Conclusion:**

Altogether, our data showed that the gene expression of PML, PML-II, and type II IFN considerably diminished in rotavirus-infected samples compared to the negative control.

## INTRODUCTION

ROtavirus is an RNA virus discovered during a three-month survey in Melbourne and diagnosed as the causative agent of gastroenteritis by electron microscopy^[^^1^^,^^2^^]^. The virus genome is organized as ds RNA helicesz^[^^3^^]^. Using high or low multiplicity of infection, all 11 segments of the genomic dsRNA occur in the cytoplasm of the rotavirus-infected cells, indicating that they are replicating at an equal rate, and assembly is supported by ATP5B as an RNA-binding protein^[^^4^^,^^5^^]^. In fact, rotavirus infection stimulates the B-cell response as the first step to eliminate the virus infection. Monocyte-derived dendritic cells present the rotavirus antigens in non-human primates and increase the expression of IL-6, IL-8, and IFN-γ^[^^6^^,^^7^^]^. Human rotavirus infection avoids the innate immune response by blocking the early IFN-I and IFN-II responses. Rotavirus stops the tumor necrosis factor-stimulated gene, namely NF-kB, which is critical for the stimulation of IFN response^[^^8^^]^.

 PMLs were firstly discovered in acute promyelocytic leukemia patients as a part of oncogenic fusion protein called PML-RAR-α. The PML gene has nine exons that undergo alternative splicing to form different PML isoforms with specific functions^[^^9^^,^^10^^]^. PML regulates the IFN response as a response to poly I:C stimulation, and PML-II inhibits the replication and the gene expression of adenovirus through the Hsp70 pathway^[^^11^^,^^12^^]^.

During the viral infection, the IFN response is triggered through the sensing viral DNA or RNA, or other products by receptors in infected or neighbor cells^[^^13^^]^. PMLs have been depicted as antiviral proteins due to the fact that they are IFN-up-regulated proteins and are the target of many RNA or DNA viruses^[^^14^^-^^17^^]^. Therefore, this study attempted to detect the virus prevalence and to analyze the effect of the infection on the innate immune response represented by PML and its regulatory effect on IFN-γ or IFN-II. 

## MATERIALS AND METHODS

**Sample collection**

Thirty-four stool and blood samples were collected from children (under 5 years) with acute diarrhea in Basrah Hospital (Basrah, Iraq) from October to December 2017. Positive or negative samples were categorized depending on the presence of rotavirus RNA in stool samples.

**RNA extraction**

RNA was extracted from stool and blood samples to detect rotavirus RNA. Gene expression profiles of PML, PML-II, and IFN-γ in rotavirus-infected children were compared with the negative samples. RNA was extracted using GENEzol^TM ^TriRNA Pure Kit from Geneaid, Taiwan, following the manufacturer’s instructions. Briefly, a suspension of stool samples (100 mg/900 µl of PBS) was spun down at 300 g for 5 min, and an equal volume of the supernatant and the lysis buffer were mixed, then the mix was transferred to the binding columns after adding ethanol at a ratio of 1:1. The columns were centrifuged at 16,000 g for 1 minute, and the filtrate was discarded. The columns were then washed twice with 400 µl or 600 µl of washing buffer 1 or 2, respectively, at 14,000 g for 1 min each. Subsequently, columns were dried by centrifugation at 14,000 g for 3 min, and RNA was eluted with 30 µl of RNase-free water, spinning down at 16,000 g for 1 min, and was kept at -80 °C. For RNA extraction from blood samples, 600 µl of the lysis buffer were added to 200 µl of the sample and mixed for a few seconds. Then the mixture was spun down for 1 min to eliminate the residues and the pure RNA was collected as described above. 

**Reverse transcription reaction**

RNAs from both stool and blood samples were converted to cDNA using Accupower RocketScript RT Premix Kit (CA: K-2101, Bioneer, South Korea). The template RNA was mixed with the reaction components (oligo(dT), free DEPC water, and reverse transcriptase), and the final volume was adjusted to 20 µl. The first cDNA strand was synthesized under conventional PCR conditions as follows: annealing at 25 °C for 10 min and the reverse transcription reaction at 45 °C for 60 min, which was heat inactivated at 95 °C for 5 min.

**Serological detection of the virus prevalence**

Rotavirus rapid test kit from Quick Profile Lumiquik (Cat 71033; Santa Clara, USA) was used to detect virus prevalence. Stool suspension (10%) from each sample was added to the sample tray and was left to diffuse. Based on the kit instruction, the rotavirus-positive sample can be identified by development of two pink lines in positions C and R (“C” here means control, and “R” means rotavirus). The same kit could detect the adenovirus in each sample, but with two pink lines in positions C and A (“A” means adenovirus). However, the negative result is shown by a single pink line only in position C, indicating that the sample does not contain adenovirus/rotavirus. 

**PCR analysis**

AccuPower® PCR PreMix Kit (CA: K-2260-4; Bioneer) was used to achieve the amplification of rotavirus NSP3 fragment according to the instructions recommended by the manufacturer. All primers used in this study are listed in Table 1. The reaction was prepared by mixing 5 µl of template cDNA, one of each NSP3 forward and reverse primer, 2 µl of PCR Master Mix, and 11 µl of free DEPC water to make a total volume of 20 μl. The PCR condition was set as : a denaturation step at 92 °C for 1 min, followed by 30 cycles of 2 s of denaturation at 92 °C, annealing at 52 °C for 15 s, an extension step at 72 °C for 32 s with a final extension at 72 °C for 3 min. PCR amplicons were electrophoresed and visualized on a 5% agarose gel.

**Real-time PCR**

Real-Time PCR reaction was prepared using AccuPower® GreenStarTM qPCR PreMix (CA: K-6210, Bioneer) according to the instructions provided by the manufacturer. The reaction mix with a total volume of 20 µl was prepared by adding 5 µl of template cDNA (concentration was adjusted to 5 ng/µl), 1.5 µl of each forward and reverse primer of PML, PML-II, and IFN-γ, and 12 µl of free DEPC water. The real- time PCR condition was set as follows: a denaturation step at 94 °C for 3 min, followed by 45 cycles of denaturation at 94 °C for 30 s, annealing at 56 °C for 30 s, and an extension step at 72 °C for 1 min with a final extension conducted at 72 °C for 5 min. The reaction was subjected to the melting curve analysis to ensure the primer specificity. 

**Table 1:** Primers were used in this study to detect the virus prevalence and cellular gene expression

**Primers**	**sequences**
NSP3^[18]^	Forward: ACCATCTACACATGACCCTC
	Reverse: GGTCACATAACGCCCC
PML^[19]^	Forward: ACACCAGTGGTTCCTCAAGCA
	Reverse: 5'-CTCGGCAGTAGATGCTGGTCA-3'
PML-II^[20]^	Forward: AGGCAGAGGAACGCGTTGT
	Reverse: GGCTCCATGCACGAGTTTTC
IFN-γ^[21]^	Forward: GTATTGCTTTGCGTTGGACA
	Reverse: GAG TGT GGA GAC CAT CAA GGA
β-actin^[22]^	Forward: CATCGAGCACGGCATCGTCA
	Reverse: TAGCACAGCCTGGATAGCAAC

## RESULTS

**Serology assay**

A serological test, using Quick Profile Lumiquik Kit (Santa Clara) was employed to detect rotavirus particles in 24 stool samples. The test relied on serological interaction with the viral particles in stool samples as an antigen. The results showed that 45% of samples were positive. 

**PCR detection of rotavirus**

**Fig. 1:**
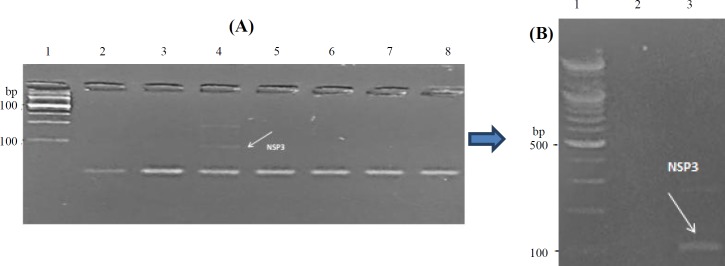
Agarose gel electrophoresis of PCR-amplified products of rotavirus species-specific primers (NSP3) using: (A) 2% agarose: lane 1, 100 bp DNA size marker; lane 2, no template (negative) control; lanes 3-8 the examined sample. (B): 5% agarose: lane 1, 100 bp DNA size marker; lane 2, no template control; lane 3, an examined sample

Molecular detection of rotavirus using a set of NSP3-specific primers amplifying 100 bp showed that only 17.6% of the samples were positive using the optimum PCR condition (Fig. 1). 

**Specificity of primers and amplification curves**

**Fig. 2:**
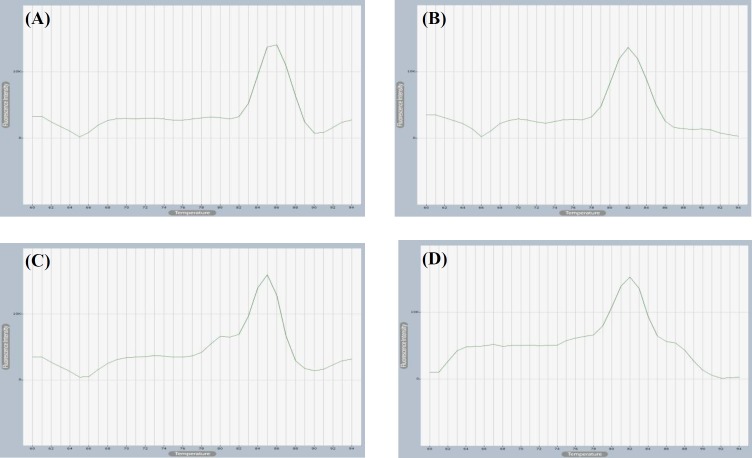
Melting curve analyses of PML, PML-II, IFN-γ, and β-actin. Fluorescence change was measured with increasing temperature to separate the ds of the DNA to form single-stranded DNA. Fluorescence stops when SYBR green dissociates from the DNA. One dissociation curve was obtained for PML (A), PML-II (B), IFN-γ (C), and β-actin (D) genes amplified by the qPCR SYBR green, which showed one peak at 82 °C, 78 °C, 82 °C, and 82 °C, respectively

**Fig. 3:**
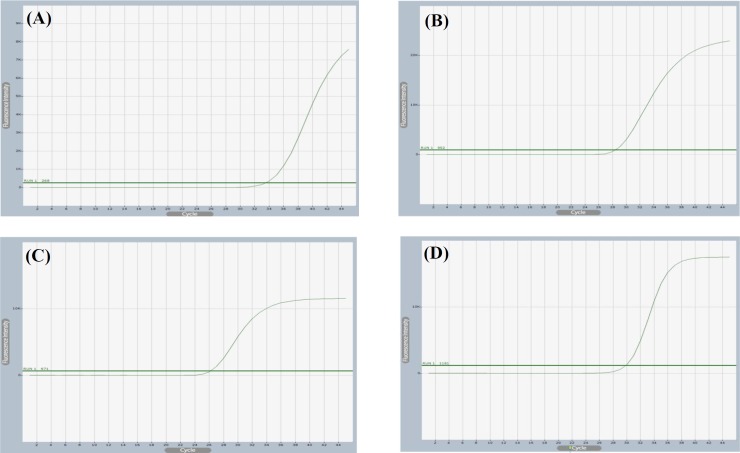
Representative amplification curves of PML, PML-II, IFN, and β-actin. Amplification curves were generated by plotting the cycle number at the crossing point with the threshold value versus the log of starting amount of template. Clear amplification curves were obtained for PML (A), PML-II (B), IFN (C), and β-actin (D), respectively

Using Exicycler^TM^ 96 Software, the melting and amplification curves were detected and carefully compared to the non-template controls, and all the PCR amplicons were subjected to dissociation curve analysis to ensure the primer specificity. Each target gene PML, PML-II, IFN-γ and β-actin the control gene showed single peak (Fig. 2). Measurable fluorescence signal from SYBR green emission was detected from PML, PML-II, IFN-γ, and β-actin reaction tubes. Although they appeared at late cycles, it was sufficient to be distinguished from the non-template control (Fig. 3). PML amplification curve was detected using PML qPCR specific primers (Fig. 3A). In order to detect which isoform of PML proteins is responsible for counteracting antiviral response against rotavirus infection, PML-II expression was measured using specific primers, the amplification curve was investigated and the threshold was set automatically (Fig. 3B). PML-related genes have been well-connected to PML functions in literatures, and it has been mentioned that there is a cross-talk between ISG and PML^[^^11^^]^. Therefore, the amplification curve of IFN-γ- was detected using specific primers (Fig. 3C), While all the relative expression was assessed by subtracting β-actin CT value which again showed an amplification curve using β-actin specific primers (Fig. 3D). in all cases, the gene expression profile was calculated based on on ∆∆CT analysis.

**PML relative expression in rotavirus-infected samples**

To evaluate the PML gene expression as a response to rotavirus infection, RNAs extracted from blood samples were used as templates for cDNA preparations. cDNA template was then used in qPCR relative expression assay (SYBR green). Apparently, the expression level of PML was found to be significantly higher in negative samples detected by either molecular analysis or serological test or by both approaches when compared to the positive samples. The fluorescence signal of PML lagged more or less five cycles behind the negative sample signal.

**Fig. 4:**
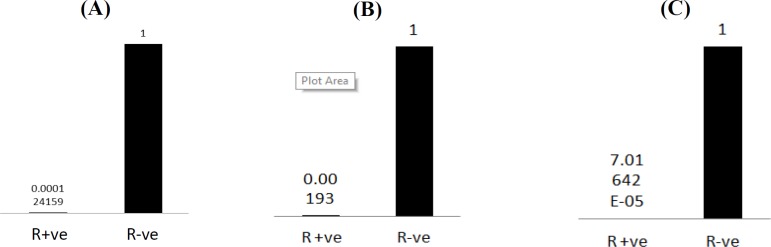
RT and real-time PCR analysis of PML (A), PML-II (B), and IFN-γ (C) mRNA relative expression in positive and negative samples. Transcript levels were quantified in cDNAs obtained from positive or negative samples using the expression of β-actin as an internal control. Relative expression assay was achieved using SYBR green as intercalating dye. Obtained data were analyzed by ΔΔCTs and then normalized to the control samples, which were equalized to 1

The value of β-actin amplification was subtracted from PML value of each sample. The ∆∆CT analysis was used here by subtracting the resultant value of the control samples from that of the infected sample. The data were then analyzed by normalizing the negative control samples to 1. Then the number of folds difference in the PML expression was calculated. Expression of PML in positive samples was roughly less than 10,000fold compared to its value in the negative control samples (Fig. 4A).

**PML-II gene expression**

Given the important role of PML-II in regulating the IFN response and its negative role in adenovirus lifecycle, the expression pattern of PML-II was examined. Again, cDNAs from the total blood RNA were made and used as templates for relative gene expression assay normalized to the control genes. In consistent with the total PML expression profile, PML-II expression was drastically reduced in rotavirus-infected samples compared to the control ones that their amplification appeared two CTs earlier. To conclude the fold change value, the negative control sample was normalized to 1 to which the positive value was compared. In fact, the PML-II expression showed a 10,000fold reduction compared to the control (Fig. 4B).

**IFN-γ expression profile**

Due to the well-known correlation between TRIM-19 family (which PML belongs to) and IFN and antiviral immune response, IFN-γ gene expression profile in both rotavirus-positive or negative samples were examined for IFN-γ mRNA expression. In consistent with the expression profile of PML, in rotavirus-infected samples, IFN-γ expression wasmassively lower than its level in the negative samples. Again, the CTs of IFN-γ in positive samples appeared six CTs behind the negative ones, and the real values of the expression were obtained by ∆∆CT analysis.

Moreover, to exactly detect the fold change, the values of IFN-γ in negative samples were normalized to 1, which showed that IFN-γ expression increased to about 40,000fold in the negative samples (Fig. 4C).

## DISCUSSION

Rotavirus infects children, particularly under five years of age^[^^23^^]^. Most of the diarrhea samples in this study were from children aged from 2-27 months, similar to studies from other Asian countries, such as Pakistan^[^^24^^,^^25^^]^. Our sample collection was conducted during autumn since rotavirus infection often increases with cold seasons and reduces during spring or summer; this is in agreement with a previous study conducted in Australia where infection with rotavirus declines in Summer^[^^26^^]^.

 The molecular detection (PCR) of rotavirus prevalence in our study showed to be 17.6%, while the serological test indicated a prevalence of 45%. Rotavirus detection by PCR was less than what was reported in Kiambu, Kenya, which reached 68.2%^[^^27^^]^. Such difference might belong to the target gene amplified in the PCR as the genes in Kenya study were VP4 and VP7, while, in this study, NSP3 was the specific amplicon. The other reason for this discrepancy might be due to the larger number of population studied since the number of samples surveyed in Kenya was 85 with 58 positive samples, but the total number of samples in the present study was 34 with only six positive cases. However, in future, using a larger size of samples is strongly recommended that might give better insights into the incidence of infection and how frequent is each type of rotavirus in affected children. The serological test in our investigation showed a higher prevalence percentage compared to other studies, which could be due to the differences in the serological methods employed in each study; ELISA was used as a detection tool in other studies^[^^27^^]^. Another reason is the cross-reactivity that might be generated due to the presence of anti-adenovirus antibodies in the same pad that also contains anti-rotavirus antibodies in Quick Profile Limiquik serological reaction.

Once IFN-γ is stimulated in the virus-infected cells, it attaches to a specific receptor in the surface of the neighboring (uninfected) cell. Consequently, STAT1 is phosphorylated and both types (STAT1-STAT2) bind to translocate from cytoplasm to nucleus and are recruited into the IFN-γ promoter activation site to induce several ISGs^[^^28^^]^.

Our results showed that IFN-γ decreases in the positive samples of rotavirus in contrast to the negative samples, which is mainly due to the developed mechanisms established by many viruses to confront the innate immunity, in order to replicate efficiently inside the cell without any restriction by the body defenses, such as IFNs. In line with our finding, Hakim *et al.*^[^^29^^]^, who conducted his study in murine models using IFN-I (α, β) and IFN-II (γ) as a therapy against rotavirus, failed in defense and giving resistance against the invaded rotavirus. These results proved that IFN-I and -II have a secondary role in controlling rotavirus infection in mice and also suggested that IFN-III (IFN-λ) is advantageous in mice models and is inhibited by the virus in intestinal epithelial cells^[^^29^^-^^31^^]^, which gives a better resistance against rotavirus. It has also been revealed that the gene expression of IFNs is down-regulated by rotavirus infection^[^^32^^]^; a rotavirus developed mechanism impedes STAT-Y701 phosphorylation with IFN overexpression^[^^33^^]^. In HT29 cells, STAT-Y701 is phosphorylated as a response to exogenous IFNs such as IFNs I, II, and III; furthermore, infected HT29 cell with simian RRV RV strain or Porcine SB1-A showed a decline in the level of IFN-γ1 receptor gene expression occurring 6-8 h post infection with these two viruses.

The IFN stimulation step is restricted by viral proteins. These viral proteins have influence on the activation of certain transcription factors in the cytoplasm of the infected cells. Rotavirus has a protein, called nonstructural protein NSP1,that is able to inhibit the IFN production. This protein can restrict IFN-α, β production by proteasome-dependent degradation through proteasome transcription factors such as IRF3, IRF7, and IRF5. NSP1 can also restrict activation of NFkB. IRF3, IRF7, and IRF5, and NF-kB are the main factors that promote the IFN production^[^^34^^]^. Similarly, it has been demonstrated that IFN is produced when^[^^35^^]^ NSPI of rotavirus destroys the IRF3. In addition to the restriction effect of rotaviruses on IFN production^[^^34^^]^, these viruses can inhibit STAT1 and STAT2 expression^[5]^. 

In our case, the samples were collected after the symptoms of diarrhea and dehydration started, normally at two days post infection. Progeny virus is formed after 10 to 12 hours, which is then released to infect other cells and to shed inside the lumen of the intestine^[^^36^^]^. Hence, the reduced expression of IFN and PML might be due to the time when the samples were collected as the virus had enough time to enter, establishing its own replication centers and synthesizing the structural proteins. This gap in time between triggering the IFN response and the appearance of symptoms is when the virus controls the cellular machinery and blocks the IFN response. In fact, the IFN response is triggered at two hours post infection, reaching the maximum at six hours post infection^[^^37^^]^; therefore, time is a critical factor here. PML expression has an inhibitory effect on the virus lifecycles, making it sensitive to IFN response and restricting its growth. For example, in case of influenza A the viral replication is reduced with the overexpression of PML and recovered with the removal of PML^[^^38^^]^. PML expression, especially I-IV, increases when IFN-γ induces STAT1phosphorylation to induce the ISG. PML knockdown will reduce the phosphorylation of STAT1 induced by IFN-γ; as a consequence, it decreases the levels of ISG transcription^[^^39^^]^.

Our results displayed that PML-II gene expression decreased in positive samples, and this might be due to the reduction in the level of gene expression of IFN-γ and total PML. As was mentioned by^[^^28^^]^, IFN-γ production in a cell infected by virus will stimulate the neighboring cell during JAK-STAT signaling and hundreds of ISGs are activated. Up-regulated PML gene expression by IFN-I or -II increases the expression of PML-II, while decrease in the PML level will certainly decrease the PML-II expression. There are many cytokines that stimulate IFN-γ-II; for example, IL-12, IL-2, IL-18, IL-15, and IFN-I (α, β). All these cytokines are critical to the stimulation of IFN-γ in natural killer cells. Therefore, IFN-I (α, β) has a significant role in stimulating IFN-γ, and inhibiting the expression of PML-II results in down-regulationn of IFN-β and^[^^40^^-^^42^^]^ Hence, the increased level of gene expression of IFN-β was noticed when PML-II expression was augmented. Similarly, when PML-II levels are reduced, IFN promoter activity is reduced to 25% compared to the control ^[^^8^^]^. In conclusion, it could be a chain of events because IFN-β reduction lowers PML-II expression and as a consequence expression of IFN-γ (which is IFN I- regulated gene) is reduced. Accordingly, in the positive samples the production of IFN-γ would be affected by IFN-1, such as IFN-β, and reduction in gene expression of IFN-γ in turn decreased the gene expression of PML-II.

## CONFLICT OF INTEREST.

None declared.
